# Book reviews

**DOI:** 10.1155/S1463924688000215

**Published:** 1988

**Authors:** Ray G. Lidzey

**Affiliations:** 6 The Spinney London N21 UK


### Book reviews

**Introduction to Computer-assisted Experimentation**
By Kenneth I. Ratzlaff (John Wiley & Sons, Inc., 1987).

This book is described in the preface as being suitable for a reader who has some familiarity with a scientific laboratory, an understanding of electricity and some experience in programming a computer. It also states that it employs a limited amount of jargon. This has resulted in a very readable book that starts with an introduction to computers and moves through computer interfacing to the use of computers in laboratory experimentation.

Careful descriptions outline the digital operation and language options. Types of interface are dealt with but are rather brief in examples of program coding practice.

A section on analogue electronics discusses operational amplifiers and filters in the signal conditioning stage preparatory to computer input. A brief chapter on digital electronics is followed by two chapters on transducers, the later terminating with a short section on laboratory robotics. Of rather more use is a chapter on data communications. The last three chapters discuss graphical presentation, computational techniques and the overall task.

Altogether this book has a lot of ground to cover and is often too brief to explain the diagramatical representations fully to a newcomer. There is a shortage of examples, little or no bibliography in some chapters, and a lack of appendices.

It may also be said that there are a lot of standard interfaces available now and building one's own can only be jusitified as a learning process.

**Ray G. Lidzey**
6 The Spinney, London N21

*Laboratory Robotics: A Guide to Planning Programming and Applications*
By W. Jeffrey and James W. Mortimer (Published by VCH Publishers, Inc., 303 N.W. 12th Avenue, Deerfield Beach, Florida 33442-1705, USA, 1987).

This slim volume is an introduction to the American scene in robotics but concentrates on the Zymate robot for most of its detailed descriptions.

Two chapters, respectively, deal with the need and justification for laboratory robots in perhaps an overly non-critical and superficial manner. It is unlikely that robots would be used 24 hours per day and seven days per week without an overseer in attendance. Generally, reliability of robot systems operating complex programs are not sufficiently fool-proof enough to be able to leave them to function without failure over long periods and, indeed, very few systems operate overnight unattended. Also the suggestion that multiple tests can be catered for by just changing the equipment layout and running another program is usually unachievable in practice. Most operators leave the equipment layout carefully undisturbed when a method is running reliably. A discussion on costing is of limited value to UK readers as prices are indicated in dollars and salaries and overheads for technical personnel would be different in Britain.

Another chapter lists applications with references confined mostly to the Zymark robot but will give a useful indication to newcomers of the range of activities laboratory robots are used on in the USA.

Some of the questions asked prior to the purchasing of a robot are presented but a lot of gaps are left particularly in the technical area.

A type of FORTH language is used to program the Zymate and illustrations are given of its use. PERL, the Perkin-Elmer language, is also touched upon.

There are a reasonable number of photographs and diagrams in the volume but the glossary is pointless. No mention is made of Sample Processors such as the Gilson or Tecan product which might be expected to fall under the umbrella description of laboratory robotics.

**Ray G. Lidzey**
6 The Spinney, London N21

**SHORT COURSES**
Loughborough University of Technology, UK, has announced the folowing short courses for 1988:

Analytical Plasma Spectrometry – 5–9 September 1988. Fee £450.

Atomic Absorption Spectrometry – 12 September 1988. Fee £70.

Modern Electroanalytical Methods – 14–16 September 1988. Fee £305.

Microcomputers in the Laboratory – 19–23 September 1988.

*Further details from Mrs J. Stirling, Department of Chemistry, Loughborough University of Technology, Loughborough, Leicestershire LE11 3TU, UK.*

